# Comparison of the effects of monounsaturated fatty acids and polyunsaturated fatty acids on the lipotoxicity of islets

**DOI:** 10.3389/fendo.2024.1368853

**Published:** 2024-03-04

**Authors:** Wen Liu, Min Zhu, Jingyi Liu, Shan Su, Xin Zeng, Fudong Fu, Yanrong Lu, Zhiyong Rao, Younan Chen

**Affiliations:** ^1^ Department of Clinical Nutrition and Key Laboratory of Transplant Engineering and Immunology, Regenerative Medicine Research Center, West China Hospital, Sichuan University, Chengdu, China; ^2^ Institutes for Systems Genetics, Frontiers Science Center for Disease-related Molecular Network, West China Hospital, Sichuan University, Chengdu, China; ^3^ Department of Clinical Nutrition, West China Hospital, Sichuan University, Chengdu, China

**Keywords:** obesity, insulin resistance, unsaturated fatty acids, saturated fatty acids, lipotoxicity

## Abstract

**Background:**

Monounsaturated fatty acids (MUFAs) and polyunsaturated fatty acids (PUFAs) have been reported to combat saturated fatty acid (SFA)-induced cellular damage, however, their clinical effects on patients with metabolic diseases such as diabetes and hyperlipidemia are still controversial. Since comparative studies of the effects of these two types of unsaturated fatty acids (UFAs) are still limited. In this study, we aimed to compare the protective effects of various UFAs on pancreatic islets under the stress of SFA-induced metabolic disorder and lipotoxicity.

**Methods:**

Rat insulinoma cell line INS-1E were treated with palmitic acid (PA) with or without UFAs including eicosapentaenoic acid (EPA), docosahexaenoic acid (DHA), arachidonic acid (AA), and oleic acid (OA) to determine cell viability, apoptosis, endoplasmic reticulum (ER) stress, and inflammatory. In vivo, male C57BL/6 mice were fed a 60% high-fat diet (HFD) for 12 w. Then the lard in HFD was partially replaced with fish oil (FO) and olive oil (OO) at low or high proportions of energy (5% or 20%) to observe the ameliorative effects of the UFA supplement.

**Results:**

All UFAs significantly improved PA-induced cell viability impairment in INS-1E cells, and their alleviation on PA induced apoptosis, ER stress and inflammation were confirmed. Particularly, OA had better effects than EPA, DHA, and AA on attenuating cellular ER stress. In vivo, the diets with a low proportion of UFAs (5% of energy) had limited effects on HFD induced metabolic disorder, except for a slight improved intraperitoneal glucose tolerance in obese mice. However, when fed diets containing a high proportion of UFAs (20% of energy), both the FO and OO groups exhibited substantially improved glucose and lipid metabolism, such as decrease in total cholesterol (TC), low-density lipoprotein (LDL), fasting blood glucose (FBG), and fasting blood insulin (FBI)) and improvement of insulin sensitivity evidenced by intraperitoneal glucose tolerance test (IPGTT) and intraperitoneal insulin tolerance test (IPITT). Unexpectedly, FO resulted in abnormal elevation of the liver function index aspartate aminotransferase (AST) in serum. Pathologically, OO attenuated HFD-induced compensatory hyperplasia of pancreatic islets, while this effect was not obvious in the FO group.

**Conclusions:**

Both MUFAs and PUFAs can effectively protect islet β cells from SFA-induced cellular lipotoxicity. In particular, both OA in vitro and OO in vivo showed superior activities on protecting islets function and enhance insulin sensitivity, suggesting that MUFAs might have greater potential for nutritional intervention on diabetes.

## 1 Introduction

Obesity is one of the major risk factors for development impaired glucose tolerance (IGT), type 2 diabetes (T2D), and cardiovascular disease (CVD) (1–3). T2D is a multifactorial disease influenced by genetics, environment, diet, and others. Dietary fat is an essential part of the daily diet, and diets rich in saturated fatty acids (SFAs) greatly contribute to the development of obesity and T2D (4). Excessive dietary intake of saturated fatty acids, or enhanced lipolysis in adipose tissue due to peripheral insulin resistance, can result in an increase in free fatty acids in the peripheral circulation and subsequent ectopic deposition in nonadipose tissues, causing lipotoxic damage to multiple tissues and organs (5). SFAs, such as palmitic acid (PA) and stearic acid (SA), are the major dietary SFAs that are demonstrated able to cause cellular lipotoxicity. Excessive uptake of them are prone to induce endoplasmic reticulum (ER) stress, oxidative stress, mitochondrial dysfunction, impaired autophagy, and chronic inflammation attributable to an increase in the accumulation of lipid droplets and toxic metabolites such as ceramides, leading to an imbalance of cellular homeostasis, an increase in cellular stress, and ultimately cell dysfunction or cell death. In hepatocytes and adipocytes, SFAs cause disorders of glucose-lipid metabolism and induce peripheral insulin resistance (IR). In the pancreas, SFAs inhibit the synthesis and secretion of insulin by pancreatic β cells and promote β-cell apoptosis, leading to a decrease in the number and function of β cells, and thus contributing to the development of diabetes mellitus. Additionally, because pancreatic β cells are responsible for the synthesis of insulin to regulate overall blood glucose homeostasis, they are also intolerant to protein misfolding stress in the ER, making them one of the most vulnerable tissues to oxidative stress and ER stress, two important lipotoxic pathological processes. Our previous studies have shown that SFA treatment of INS-1E cells induces ER stress and apoptosis (6). In addition, it was reported that SFAs activates JNK through the activation of inositol-requiring protein 1α (IRE1α) and further through phosphorylation modification. JNK inhibit intracellular anti-apoptotic molecules through regulation of the Bcl-2 family or BAX, thereby inducing mitochondria-mediated apoptosis (7, 8). Moreover, lipotoxicity induces β-cell death via the PKC signaling pathway, MAPK signaling pathway, Akt signaling pathway, and NLRP3 inflammasome (9).

Piles of evidence has suggested that monounsaturated fatty acids (MUFAs) and polyunsaturated fatty acids (PUFAs) are able to ameliorate SFA-induced cellular damage, and are beneficial for reducing the risk of various metabolic disorders such as T2D, cardiovascular disease (CVD), and nonalcoholic fatty liver disease (NAFLD) (10, 11). The Global Burden of Disease Study also recommends reducing saturated fat intake and replacing saturated fats with polyunsaturated fats for diabetes prevention (12).

N-3 PUFAs, with the first double bond located on the third carbon atom at the methyl end of the fatty acid chain, are the most widely studied PUFAs, particularly eicosapentaenoic (EPA, 20:5) and docosahexaenoic acid (DHA, 22:6). It is widely believed that EPA and DHA regulate energy metabolism, and have advantages on anti-inflammation, promoting insulin secretion, and enhancing peripheral insulin sensitivity, resulting in reduced risk of a variety of metabolic diseases such as CVD, T2D, hyperlipidemia, and even cancer (13, 14). According to a meta-analysis of thirty clinical studies, N-3 PUFA supplementation significantly affects fasting blood glucose (FBG) and insulin resistance in diabetic patients, suggesting that supplementation with N-3 PUFAs has a protective potential on diabetes (15).

Oleic acid (OA, 18:1), which has a double bond located on the ninth carbon atom at the methyl end of the fatty acid chain, is the most physiologically concentrated monounsaturated fatty acids in the body (16). Studies have shown that similar to N-3 PUFAs, OA also can regulate metabolic diseases, owning to its anti-inflammatory and antioxidant properties, as well as effects on reducing DNA damage, promoting insulin secretion, and improving insulin resistance (17). Clinical studies have suggested that the MUFA diet is effective at reducing HbA1c in diabetic patients and is recommended for use in the dietary regimen for T2D (11) A study of the relationship between fatty acids and the risk of IGT and T2D in U.S. adults suggested that four unsaturated fatty acids (octadecenoic acid (18:1, 18:2, and 18:3) and eicosenoic acid (20:1)), found in a high percentage of natural fats and oils, may reduce the risk of T2D (18).

Although the modulatory effects of UFAs on metabolic diseases have been widely recognized internationally, the effects of different UFAs on ameliorating cellular lipotoxicity have been reported in the literatures with varying and even contradictory results. Therefore, in this study, we aimed to investigate the effects and mechanisms of different PUFAs and MUFAs in improving the SFA-induced lipotoxicity of pancreatic islet β cells in an *in vitro* model in the rat insulinoma cell line INS-1E stimulated with SFAs, and in an *in vivo* mouse model of high fat diet (HFD)-induced obesity with impaired glucose tolerance, to compare the effects of diets with high and low doses of fish oil (FO) or olive oil (OO) on overall insulin sensitivity and glucose-lipids metabolism.

## 2 Materials and methods

### 2.1 Reagents and antibodies

Palmitic acid (PA) was purchased from Aladdin (Shanghai, China). Oleic acid (OA), eicosapentaenoic acid (EPA), docosahexaenoic acid (DHA), arachidonic acid (AA), and stearic acid (SA) were purchased from Sigma (Shanghai, China). Bovine serum albumin (BSA) was purchased from Solarbio (Beijing, China). A Cell Counting Kit-8 (CCK-8) was purchased from Beyotime (Shanghai, China). Anti-Cleaved caspase-3, anti-IRE1α antibody, anti-p-eif2a antibody, and anti-BAX antibody were purchased from Cell Signaling Technology (Hong kong, China). Donkey Anti-Mouse IgG H&L (Alexa Fluor^®^ 488), Donkey Anti-Rabbit IgG H&L (DyLight^®^ 550), and anti-BCL-2 antibody were purchased from Abcam (Cambridge, UK). Anti-ATF6 antibody and anti-CHOP antibody were purchased from Affinity bioscience (Jiangsu, China). Anti-actin antibody was purchased from ABclonal (Shanghai, China). Anti-BIP antibody and anti-glucagon antibody was purchased from absin (Shanghai, China). Anti-Insulin antibody and anti-ATF4 antibody were purchased from Huaan (Hangzhou, China). Anti-XBP-1 antibody and HRP labelled goat anti-rabbit IgG were purchased from Wanlei (Shenyang, China). TRIizol reagent was purchased from Ambion (TEXAS, USA). A high-capacity cDNA synthesis kit was purchased from Vazyme (Nanjing, China).

### 2.2 Preparation of fatty acid solutions

Fatty acid was dissolved in 100% ethyl alcohol to produce a highly concentrated solution of 100 mM, which was subsequently bound with 20% fatty acid-free BSA by incubation at 50°C for 1 h to yield a final stock solution of 10 mM. The 200 µM fatty acid working solution has a concentration of 54 µM BSA and 2% (v/v) alcohol.

### 2.3 Cell culture

The rat insulinoma cell line INS-1E (purchased from ATCC) was cultured in RPMI 1640 (HyClone, Utah, USA) supplemented with 10% fetal bovine serum (FBS), 1 mM sodium pyruvate and 50 μM β-mercaptoethanol.

### 2.4 Cell viability

The cells were cultured in a 96-well plate overnight at a density of 5000 cells/well after the treatments, then the cells were washed three times with PBS and cell viability was measured using a Cell Counting Kit-8, according to the manufacturer’s instructions.

### 2.5 Western blot analysis

The protein extracts were separated via 10% SDS−PAGE and transferred to 0.2 μM PVDF membranes. After being blocked for 2 h in 5% skim milk at room temperature, the membranes were subsequently probed with primary and secondary antibodies. The immunoblots were visualized using a ChemiDoc™ imaging system (Bio-Rad, USA).

### 2.6 RNA isolation and real-time PCR

Total RNA was reverse-transcribed into cDNA using a high-capacity cDNA synthesis kit. Real-time PCR was performed to assess gene expression on a Bio-Rad qPCR machine (CFX96, Bio-Rad, USA) using SYBR Green master mix (Vazyme, China). The sequences of primers are summarized in **Table 1**.

**Table 1:** Primer sequence for real-time PCR.

Gene	Sequence
R-*actin*-F	CTGGGACAGCAGCCTGTATT
R-*actin*-R	CGCTAGCCCTTGACTCTCTC
R-*Bip*-F	CAAGAACCAACTCACGTCCA
R- *Bip* -R	AACCACCTTGAATGGCAAGA
R-*Chop*-F	CGGGTGGCAGCGACAGAG
R- *Chop* -R	CAGGTGTGGTGGTGTATGAAGATG
R-*Grp94*-F	CTGATGATGAAGCCGCAGTA
R- *Grp94*-R	GCCTCTGCCATATTGGTTTG
R-*Fkbp11*-F	TACACTACACGGGCAGCTTG
R- *Fkbp11*-R	CAGAAGGCTCTGCTCCAGAC
R-*Tnfα*-F	GCTCCCTCTCATCAGTTCCA
R- *Tnfα*-R	GCTTGGTGGTTTGCTACGAC
R-*Il6*-F	ACTTCCAGCCAGTTGCCTTCTTG
R- *Il6*-R	TGGTCTGTTGTGGGTGGTATCCTC

### 2.7 Animals and treatments

Male C57BL/6 mice (6- to 7-week-old) were purchased from ENSIWEIER Animal Company (Chengdu, China), and housed in animal care facilities under controlled temperature (21 - 25°C) and humidity (40 - 70%) with a 12 h light and dark cycle. Mice were randomly divided into 2 groups: the CHOW group and the high-fat diet (HFD) group. After 12 or 16 weeks, the HFD group was divided into three groups: the HFD group, the HFD+FO (H+F) group (fish oil, By-Health, China), and the HFD+OO (H+O) group (olive oil, Ouwei Li, China). A 60% HFD was purchased from Jiangsu Synergy Company (Jiangsu, China), and customized fish oil supplement (H+F) and olive oil supplement (H+O) feeds were made by the same company by replacing 20% or 5%, respectively, of the high-fat diet according to **Table 2** (5%) and **Table 3** (20%). Low (5%) - and high-dose (20%) experimental interventions are shown in **Supplementary Figures 1**, **2**. Body weight was monitored every week, and food intake was recorded. The mice were sacrificed after an overnight fast, and blood and pancreatic tissue samples were collected. The experimental procedures were approved by the Institutional Animal Care and Use Committee (IACUC) of Sichuan University (Approval number: 20220119002).

**Table 2:** Low-dose fish oil/olive oil high-fat diet.

		60%HFD		H+ F (5%)		H+O (5%)	
		mass ratio (%)	energy ratio (%)	mass ratio (%)	energy ratio (%)	mass ratio (%)	energy ratio (%)
Protein		23.25	18.14	23.25	18.14	23.25	18.14
Fat	Lard oil	34.55	60.64	31.7	55.48	31.7	55.48
	Fish oil	0		2.85	5	2.85	5
	Olive oil	0		0	0	11.4	0
Carbs		27.2	21.22	27.2	21.22	27.2	21.22

**Table 3:** High-dose fish oil/olive oil high-fat diet.

		60%HFD		H+ F (20%)		H+O (20%)	
		mass ratio (%)	energy ratio (%)	mass ratio (%)	energy ratio (%)	mass ratio (%)	energy ratio (%)
Protein		23.25	18.14	23.25	18.14	23.25	18.14
Fat	Lard oil	34.55	60.64	23.15	40.64	23.15	40.64
	Fish oil	0		11.4	20	0	20
	Olive oil	0		0	0	11.4	0
Carbs		27.2	21.22	27.2	21.22	27.2	21.22

### 2.8 Biochemical analysis

Fasting blood glucose (FBG), aspartate aminotransferase (AST), triglyceride (TG), total cholesterol (TC), high-density lipoprotein cholesterol (HDL-C), and low-density lipoprotein cholesterol (LDL-C) were detected by an autoanalyzer (Cobas 6000 c501, Roche Diagnostics, Switzerland).

### 2.9 Fasting blood insulin detection

The FBI levels of the mice were measured using an ELISA kit (Mercodia Ultrasensitive Mouse Insulin ELISA, Sweden).

### 2.10 Glucose tolerance test and insulin tolerance test

To perform a GTT, the mice were fasted overnight for 16 h, after that 2 g/kg body weight glucose (Kelun, Sichuan, China) was injected intraperitoneally. Blood glucose was measured at 0, 15, 30, 60, and 120 min after injection. Glucose concentrations were monitored using a precalibrated glucometer (Accu-Chek Aviva, Roche, Basel, Switzerland). The area under the curve (AUC) was assessed for each group from 0 to 120 min post glucose injection. To perform an ITT, the mice were fasted for 6 h, and 0.75 U/kg body weight insulin (Wanbang, Jiangsu, China) was injected intraperitoneally. The following steps are the same as those for the GTT.

### 2.11 Hematoxylin and eosin staining

After the mice were sacrificed, the pancreatic tissue samples were fixed in a 10% formaldehyde solution and embedded in paraffin. Then, the paraffin sections were dewaxed and rehydrated in differential alcohol gradients for subsequent hematoxylin and eosin (HE) staining to observe histopathological changes via standard light microscopy.

### 2.12 Immunofluorescence staining

After dewaxing, the sections were blocked in goat serum for one hour, incubated with anti-insulin antibody (1:500) or anti-glucagon antibody (1:200) at 4°C overnight, washed with PBS 3 times, incubated with a fluorescent secondary antibody at 37°C for 1 hour, washed with PBS 3 times, and sealed with fluorescent tablets containing DAPI.

### 2.13 Statistical analysis

Histopathological data were analyzed using ImageJ software, and all cellular and animal pathological data are expressed as the means ± SDs. Statistical analysis was performed using GraphPad Prism 8.0. Student’s t test was used to compare two groups, and one-way ANOVA was used for the analysis of multiple groups. *P*<0.05 was considered to indicate statistical significance.

## 3 Results

### 3.1 UFAs improve SFA-induced cell viability damage

**Figure 1:**
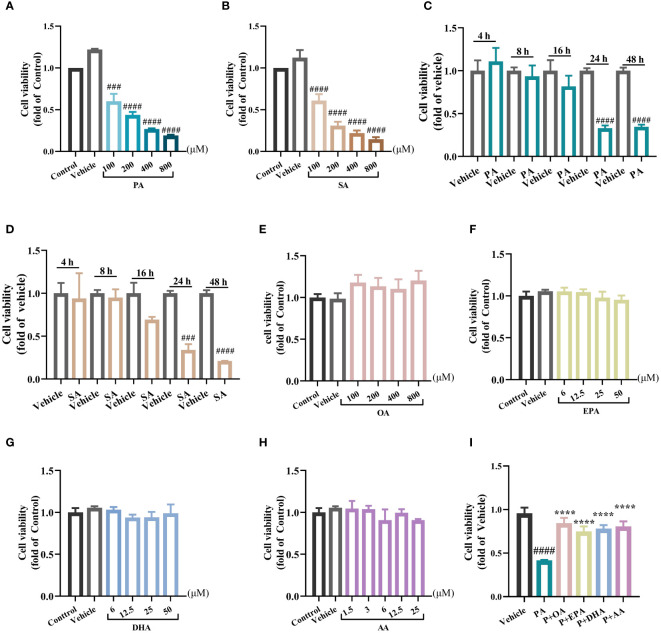
MUFAs/PUFAs improve the cell viability damage induced by SFAs in INS-1E cells. **(A)** Effects of 24 h treatment of PA (100 μM~800 μM) on the viability of INS-1E cells. **(B)** Effects of 24 h treatments of SA (100 μM~800 μM) on the viability of INS-1E cells. **(C)** The cell viability of INS-1E treated with 100 μM PA at different time points. **(D)** The cell viability of INS-1E treated with 100 μM SA at different time points. **(E)** Effects of 24 h treatment of OA (100 μM~800 μM) on the viability of INS-1E cells. **(F)** Effects of 24 h treatment of EPA (6 μM~50 μM) on the viability of INS-1E cells. **(G)** Effects of 24 h treatment of DHA (6 μM~50 μM) on the viability of INS-1E cells. **(H)** Effects of 24 h treatment of AA (1.5 μM~25 μM) on the viability of INS-1E cells. **(I)** Effects of 200 μM OA, 25 μM EPA, DHA, and AA combined with 200 μM PA respectively, on the viability of INS-1E cells. ^##^ *P*<0.01, ^###^ *P*<0.001, ^####^ *P*<0.0001 vs. Control/Vehicle group. ^****^ *P <*0.0001 vs. PA group. Data are expressed as mean ± SD, (n≥3).

Firstly, we examined the toxicity of saturated fatty acids (SFAs) on the cellular viability of the rat insulinoma cell line INS-1E by a CCK8 assay. After incubating INS1-E cells with different concentrations (100, 200, 400, or 800 μM) of the SFAs palmitic acid (PA) or stearic acid (SA) for 24 h, the cell viability decreased with increasing SFA concentration (**Figures 1A, B**). To observe the time-dependent effects of PA or SA stimulation, INS1-E cells were stimulated with 200 μM PA or SA for 4 h, 8 h, 16 h, or 24 h, respectively. The results showed that cell viability tended to decrease after 8 h and 16 h of treatments with PA or SA, but the differences were not statistically significant, and the cell viability decreased by approximately 65% after 24 h of both treatments (*P*<0.001), suggesting that cell viability decreased with prolonged treatment with SFAs (**Figures 1C, D**). Therefore, the PA stimulation with concentration of 200 μM, and the endurance of 24 h was used in subsequent experiments. Interestingly, INS-1E cells treated with unsaturated fatty acids Oleic acid (OA) (100, 200, 400, or 800 μM), eicosapentaenoic acid (EPA) (6, 12.5, 25, or 50μM), docosahexaenoic acid (DHA) (6, 12.5, 25, or 50μM), or arachidonic acid (AA) (1.5, 3, 6, or 25μM) for 24 h showed no significant impairment on cell viability (**Figures 1E-H**), suggesting the lipotoxicity was predominantly caused by SFAs but not unsaturated fatty acids (UFAs). Given that different fatty acid concentrations can vary considerably in different individuals and different disease contexts, we reviewed the approximate range of free fatty acid concentrations under normal physiological conditions. The concentration range of AA is about 3.6-8.9 μM, the concentration range of EPA is about 26.7-42.6μM, the concentration range of DHA is about 70- 113.8 μM, and the concentration range of OA is about 162-365 μM (16, 19–21). Finally, refer to the concentrations of some *in vitro* cell experiments (22, 23), we used a concentration of 25 μM for PUFAs (EPA, DHA and AA), and a concentration of 200 μM for OA, which is the same as that for PA. Next, we tested the antagonism of UFAs against SFAs. The UFAs of 200 μM OA, 25 μM EPA, (DHA, and AA) were combined with 200 μM PA, respectively; to investigate their protective effects against PA induced cell damage.

Strikingly, the combination of all the four kinds of UFAs with PA profoundly attenuated PA-induced cellular injury and restored the viability of INS-1E cell close to that of the control group, further validating the ameliorative effect of UFAs on SFA-induced lipotoxicity (**Figure 1I**).

### 3.2 UFAs improve SFA-induced cell apoptosis

**Figure 2:**
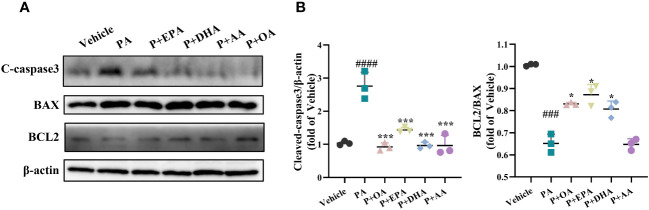
MUFAs/PUFAs ameliorate PA-induced apoptosis in INS-1E cells. **(A)** The western blots for apoptosis markers (BCL2, BAX, and cleaved caspase-3). **(B)** Statistical analysis of protein expression levels of cleaved caspase-3. ^###^ *P*<0.001, ^####^ *P*<0.0001 vs. Vehicle group. ^*^ *P <*0.05, ^***^ *P <*0.001 vs. PA group. Data are expressed as mean ± SD, (n≥3).

It is reported that SFAs can induce cell apoptosis in islet β cells, and we then examined the expression of the apoptosis-associated proteins BCL2, BAX, and cleaved caspase-3 in INS-1E cells after treatments with different fatty acids by Western blotting. The results showed that, compared with the control group, PA significantly increased the expression of cleaved caspase-3 and decreased the expression of the antiapoptotic protein BCL2 (**Figures 2A, B**). In addition, UFAs significantly reduced PA-induced cleaved caspase-3 hyperexpression. Moreover, they increased the expression ratio of BCL2/BAX, except for AA, suggesting that UFAs have great potential for alleviating apoptosis (**Figure 2B**).

### 3.3 UFAs improve SFA-induced ER stress and inflammatory gene expression

**Figure 3:**
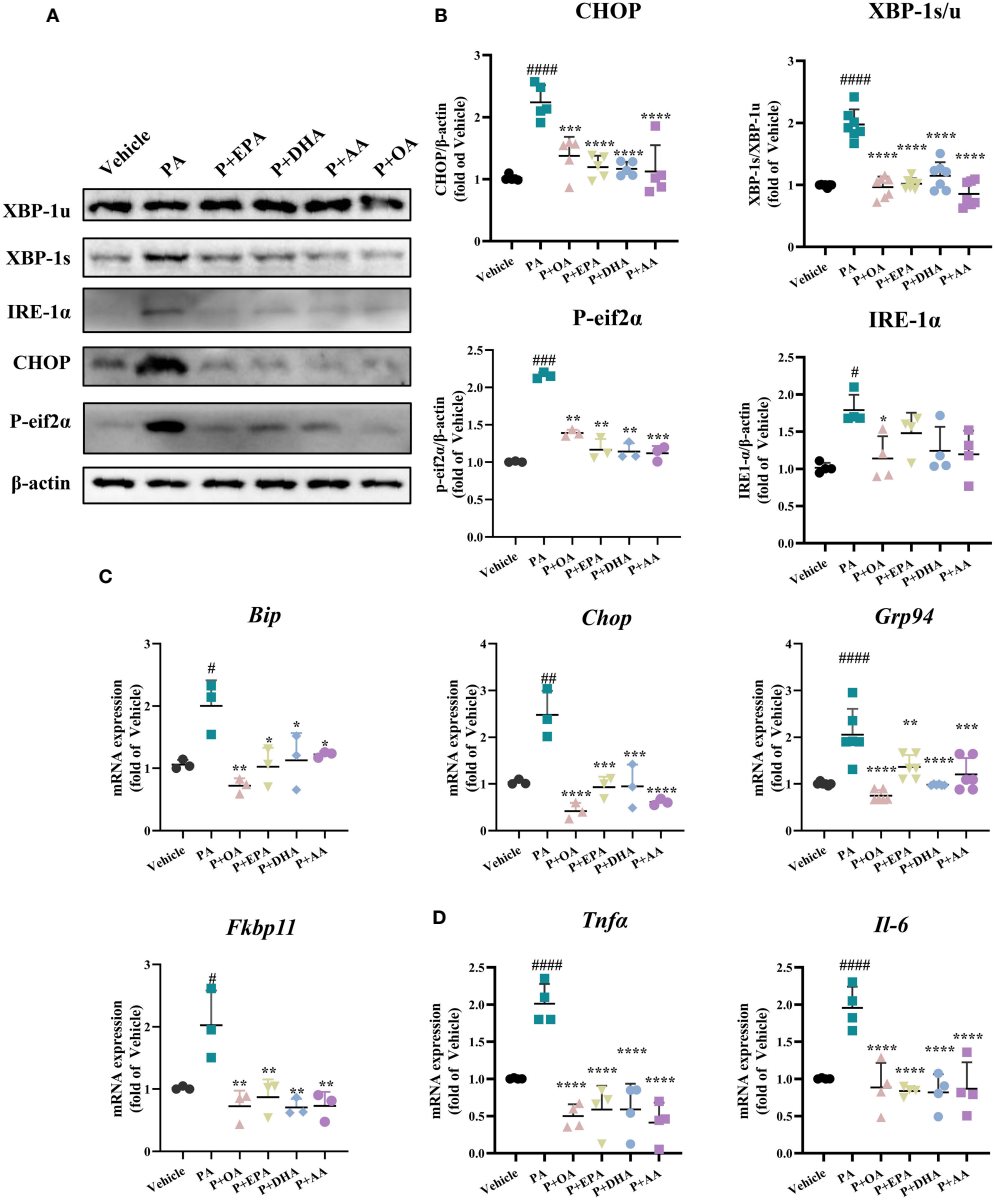
MUFAs/PUFAs ameliorate PA-induced ER stress and the expressions of inflammatory genes. **(A)** The expression of ER stress markers (CHOP, P-eif2α, XBP1, and IRE1α) were examined using immunoblots. **(B)** Statistical analysis of protein expression levels in Figure **(A)**. **(C)** The mRNA expression of ER stress markers (*Bip, Chop, Grp94*, and *Fkbp11*) was assessed via real-time PCR. **(D)** The mRNA expression of *Tnfα* and *Il-6* was assessed via real-time PCR. ^#^ *P*<0.05, ^##^ *P*<0.01, ^###^ *P*<0.001, ^####^ *P*<0.0001 vs. vehicle group. ^*^ *P <*0.05, ^**^ *P <*0.01, ^***^ *P <*0.001, ^****^ *P <*0.0001 vs. PA group. Data are expressed as mean ± SD, (n≥3).

Next, we examined the expression of endoplasmic reticulum (ER) stress-related proteins in INS-1E cells. The protein expressions of BIP, CHOP, XBP1s, XBP1u, IRE1-α, ATF4, ATF6, and P-eif2α were detected via Western blotting (**Figure 3A** and **Supplementary Figure 3A**). PA-induced ER stress in INS-1E cells was manifested by activation of the IRE1-α/XBP1 pathway and a significant increase of the expression of CHOP, but there was no difference in the expression of BIP, ATF4, or ATF6 among the groups (**Figure 3B** and **Supplementary Figure 3B**). UFAs interventions also showed that the combination of OA, EPA, DHA, and AA with PA respectively, did not affect the protein expressions of BIP, ATF4, or ATF6 but did significantly reduce the expressions of CHOP and P-eif2α and decreased the XBP-1 s/u ratio. Among the four UFAs groups, only the PA+OA group exhibited significant downregulation of IRE1-α expression. We also detected the mRNA levels of the ER stress-related genes *Bip, Chop, Fkbp11, and Grp94* by real-time PCR, and all the UFAs intervention groups exhibited significantly reduced expression levels of the above genes in comparison with that of PA group. It’s worthy to note that OA showed the greatest improvement among the four UFAs (**Figure 3C**).

Subsequently, we also examined the gene expressions of the inflammatory factors *Tnfα* and *Il-6*, and all the UFAs significantly inhibited PA-induced inflammatory gene expressions in INS-1E cells. These results suggest that both MUFAs and PUFAs can effectively ameliorate PA-induced ER stress and inflammation in INS-1E cells (**Figure 3D**).

### 3.4 A low-dose fish oil or olive oil diet did not improve IR in obese mice

C57BL/6 mice (6 weeks) were fed a HFD for 12 weeks, and insulin resistance (IR) and glucose–lipid metabolism disorders were successfully established in these obese mice (**Supplementary Figure 4**). The mice in the HFD group were then divided into 3 groups and fed with different customized diets: 1) HFD (60% fat energy); 2) H+F (fish oil, FO) diet; and 3) H+O (olive oil, OO) diet. After 5% of the energy provided by the lard in HFD was replaced with OO or FO, the mice were fed for a further 10 weeks. In the end of the experiment, the HFD, H+O, and H+F groups exhibited significantly greater body weights increase than the CHOW group, and there was no obvious difference in body weight among the three HFD groups. Moreover, there was no statistically significant difference in food intake among the groups, indicating that the low-dose UFA diet did not affect the food intake in these mice (**Table 4**). Compared with those in the HFD group, the serum total cholesterol (TC) in the H+F group was significantly lower, and the serum triglyceride (TG) level tended to decrease. In contrast, the H+O treatment did not significantly improve the blood lipid levels. These findings suggested that low dose of fish oil may have a stronger effect than low dose of olive oil on regulating blood lipids in obese mice (**Table 4**).

**Table 4:** Low-dose fish oil/olive oil high-fat diet interferes with biochemical indices of obese mice.

5%	CHOW (n=9)	HFD (n=9)	H+F (n=9)	H+O (n=9)
BW	32.092 ± 1.507	41.559 ± 6.012^##^	41.396 ± 6.588^#^	43.996 ± 4.701^#^
Food intake	3.398 ± 0.349	3.238 ± 0.494	3.113 ± 0.5	3.165 ± 0.470
HDL-C	1.824 ± 0.170	3.192 ± 0.307^#^	1.728 ± 0.980	2.4 ± 0.835
LDL-C	0.256 ± 0.072	0.828 ± 0.212^##^	0.616 ± 0.334	0.564 ± 0.140
TG	5.056 ± 1.926	7.94 ± 1.160	5.96 ± 2.584	7.928 ± 1.170
TC	2.264 ± 0.213	3.932 ± 0.381^##^	2.512 ± 1.052^*^	2.912 ± 0.828

BW, body weight; Food intake, g/mouse/day; HDL-C, High density lipoprotein cholesterol; LDL-C, Low density lipoprotein cholesterol; TG, Total triglycerides; TC, Total cholesterol. ^#^P<0.05, ^##^P<0.01vs. CHOW group. ^*^P<0.05 vs. HFD group. Data are expressed as the mean ± SD, (n≥6).

**Figure 4:**
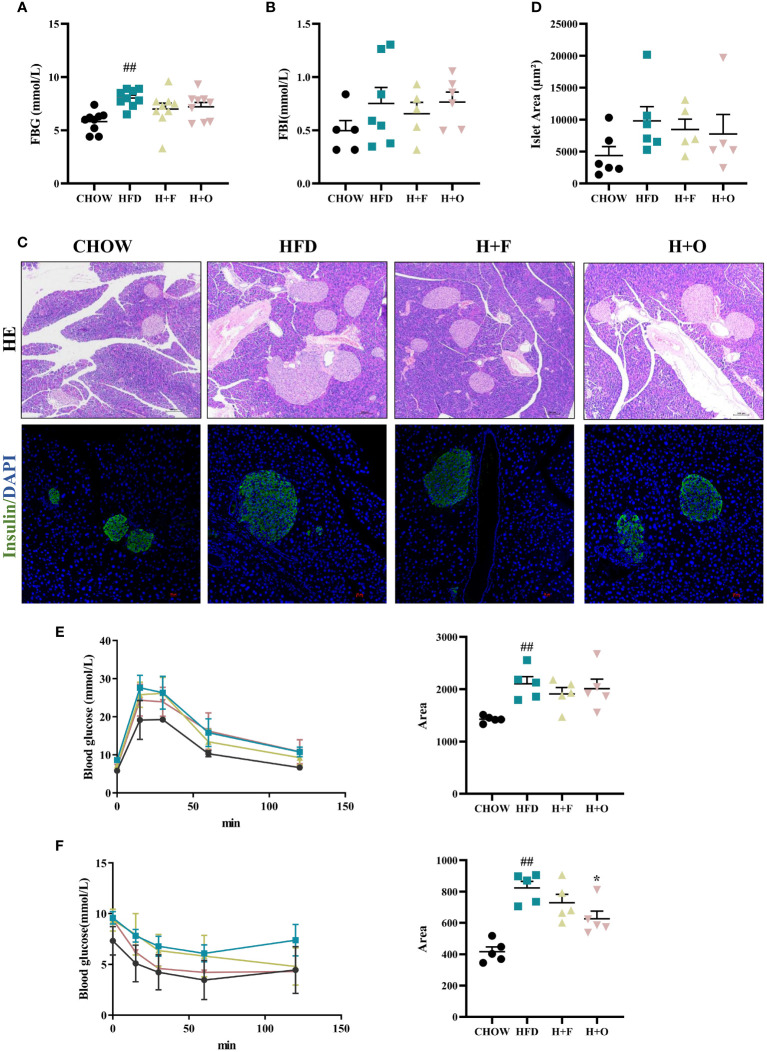
Effects of low-dose fish oil/olive oil supplement diets on HFD induced obese mice. **(A)** Fasting blood glucose (FBG). **(B)** Fasting blood Insulin (FBI). **(C)** HE staining and immunofluorescence Insulin/DAPI staining of mouse pancreatic tissues (200X). **(D)** Islet area statistics. **(E)** Intraperitoneal glucose tolerance test (IPGTT) and the area under the curve (AUC) of IPGTT. **(F)** Intraperitoneal insulin tolerance test (IPITT) and AUC of IPITT. ^##^ *P*<0.01 vs. CHOW group. Data are expressed as the mean ± SEM, (n≥5).

In terms of glucose metabolism, fasting blood glucose (FBG) was significantly elevated in the HFD group than in the CHOW group, whereas the H+F and H+O groups did not show any improvement in glycemia. Additionally, fasting blood insulin (FBI) levels were not significantly different between the groups (**Figures 4A, B**). We performed HE staining of pancreatic tissues and insulin/DAPI double staining of pancreatic islets and quantified islet areas based on the fluorescence results. The results showed that the HFD induced compensatory islets enlargement and hyperplasia, while H+F and H+O did not significantly alleviate compensatory islets hyperplasia (**Figures 4C, D**). However, the H+O diet significantly increased insulin sensitivity in obese mice, evidenced by the results of the insulin tolerance test (IPITT) (**Figures 4E, F**), suggesting olive oil might have advantage in improving insulin resistance.

### 3.5 A high-dose fish oil/olive oil high-fat diet improves IR in obese mice

In the above low-dose UFA diet intervention experiments, we observed that the OO diet had a slight ameliorative effect on IR, whereas the FO diet had a modulatory effect on blood cholesterol. We then further explored the ameliorative effects of high-dose UFA diets, in which OO and FO replaced 20% energy of the HFD. Male C57BL/6 mice (6- to 7-week-old) were divided into CHOW and HFD groups. After 16 weeks of feeding with 60% high-fat feed, the body weights of HFD group were significantly increased. Then, the HFD group was divided into three groups, and in H+F and H+O groups the lard in HFD was replaced with FO or OO (11.4 g; 20% energy supply) for a further 12 weeks. In the end of experiment, mice in the HFD group had higher body weights than mice in the H+F and H+O groups. This difference in body weight was less related to energy intake, since the HFD group ate significantly less energy than the other three groups. Blood biochemistry results showed that both the H+O and H+F groups had significantly lower serum levels of total cholesterol (TC) and low-density lipoprotein cholesterol (LDL-C) than the HFD group (**Table 5**).

**Table 5:** High-dose fish oil/olive oil high-fat diet interferes with biochemical indices of obese mice.

20%	CHOW (n=14)	HFD (n=14)	H+F (n=14)	H+O (n=15)
BW	29.692 ± 2.552	39.3 ± 6.524^####^	30.902 ± 4.247^***^	33.693 ± 6.184^*^
Food intake	3.319 ± 0.437	2.775 ± 0.532^##^	3.338 ± 0.558^**^	3.484 ± 0.364^****^
HDL	1.566 ± 0.354	2.061 ± 0.615	2.171 ± 2.845	1.536 ± 0.484
LDL	0.184 ± 0.073	0.359 ± 0.113^###^	0.255 ± 0.066^*^	0.255 ± 0.066^*^
TG	0.746 ± 0.172	1.153 ± 0.375^##^	1.054 ± 0.358	0.909 ± 0.251
TC	1.66 ± 0.368	2.346 ± 0.653^##^	1.72 ± 0.281^**^	1.725 ± 0.488^*^

BW, body weight; Food intake, g/mouse/day; HDL-C, High density lipoprotein cholesterol; LDL-C, Low density lipoprotein cholesterol; TG, Total triglycerides; TC, Total cholesterol. ^##^P<0.01, ^####^P<0.0001 vs. CHOW group. ^*^P<0.05, ^**^P<0.01, ^***^P<0.001, ^****^P<0.0001 vs. HFD group. Data are expressed as the mean ± SD, (n≥6).

**Figure 5:**
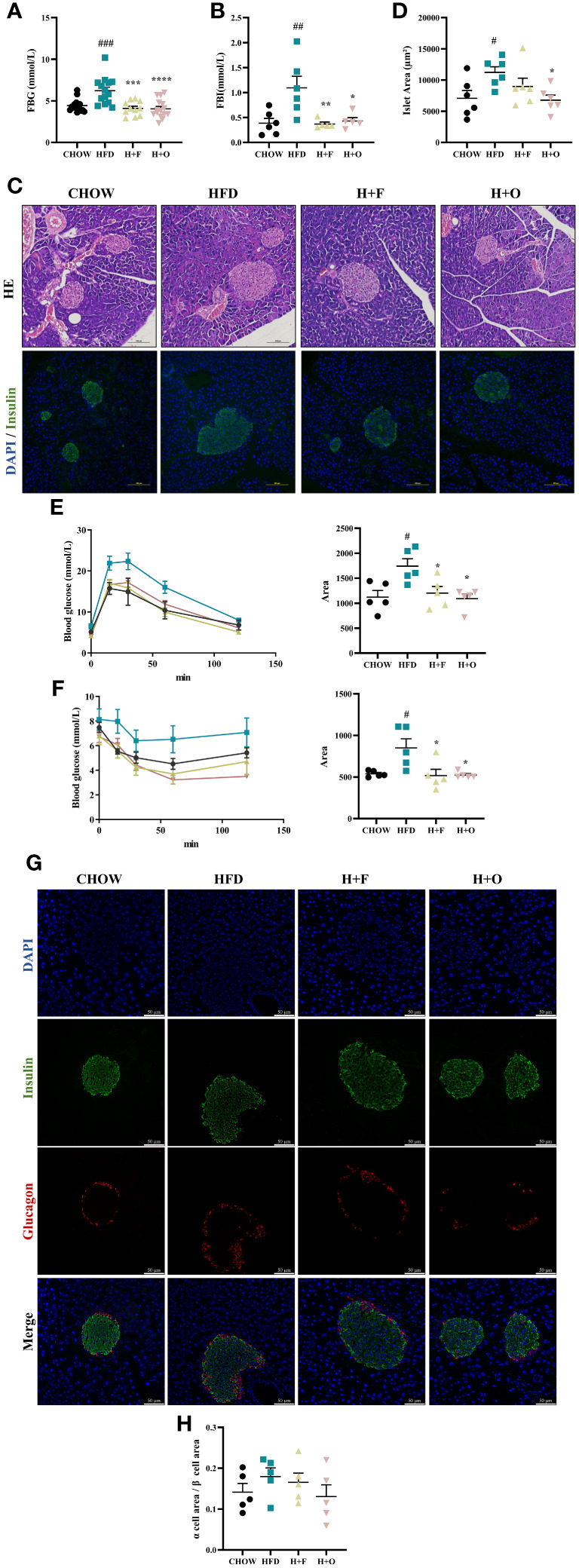
High-dose fish oil/olive oil diets improve HFD induced insulin resistance in obese mice. **(A)** Fasting blood glucose (FBG). **(B)** Fasting blood Insulin (FBI). **(C)** HE staining and immunofluorescence Insulin/DAPI staining of mouse pancreatic tissue (200X). **(D)** Islet area statistics. **(E)** Intraperitoneal glucose tolerance test (IPGTT) and the area under the curve (AUC) of IPGTT. **(F)** Intraperitoneal insulin tolerance test (IPITT) and AUC of IPITT. **(G)** Glucagon/Insulin/DAPI immunofluorescence staining of mouse pancreatic tissues. **(H)** The ration of α cell/β cell area ^#^ *P*<0.05, ^##^ *P*<0.01, ^###^ *P*<0.001 vs. CHOW group. ^*^ *P*<0.05, ^**^ *P*<0.01, ^***^ *P*<0.001, ^****^ *P*<0.0001 vs. HFD group. Data are expressed as the mean ± SD, (n≥5).

Furthermore, high-dose UFA diets for 12 weeks resulted in a significant decrease in FBG and FBI in both the H+F and H+O groups compared to those in the HFD group (**Figures 5A, B**). Based on the HE staining of pancreatic tissues, as well as Insulin/DAPI double staining of pancreatic islets, compensatory hyperplasia of pancreatic islets was induced in the HFD group. However, the size of islets was significantly restored to normal in the H+O group (**Figures 5C, D**), while the H+F group showed less changes of islets morphology in comparison to the H+O group. Moreover, according to the results of the IPGTT and IPITT, both UFA diets remarkably improved insulin sensitivity (**Figures 5E, F**). Then, we determined the α cell/β cell area ratio by triple staining with glucagon/insulin/DAPI. The α cell/β cell area ratio was slightly elevated in the HFD group, suggesting that the high-fat diet also induced an increase in α-cells mass. In contrast, the H+O group had little effect on pancreatic islet area and α/β ratio. These changes in expression were consistent with the previous changes, suggesting that both high-dose fish oil and olive oil diets have ameliorative effects on hyperlipidemia and insulin sensitivity in the context of HFD-induced obesity, while olive oil rich diet showed such advantage in maintaining the physiological function of pancreatic islets (**Figures 5G, H**).

## 4 Discussion

In our experiments, the saturated fatty acid PA impaired the viability of INS-1E cells in a time- and dose-dependent manner and induced endoplasmic reticulum (ER) stress and apoptosis. UFAs had no effect on cell viability and instead rescued PA-induced cell damage. There is strong evidence that SFA exposure impairs insulin secretion and induces apoptosis in pancreatic islet cells (24–27). Silencing stearoyl CoA desaturase (SCD), PA/SA still induces ER stress and inflammation in the human β-cell line, EndoC-bH1. Treatment with oleate or palmitoleate, products of SCD, reversed the inflammation and ER stress (28). In human embryonic stem cell-derived β cells (SC-β-cells), PA treatment results were consistent with our experimental results that PA-induced apoptosis in SC-β-cells and increased the expression of XBP1s, XBP1, and IRE1-α (29).In human islets, PA treatment for 24h (30) or 48h (31) induced increased expression of ER stress markers, whereas OA did not induce ER stress signaling in human islets. The lipotoxicity of PA on hepatocytes, and muscle cells has also been shown in other published reports. In the hepatocellular carcinoma cell line HepG2, cell viability decreased with increasing concentrations of PA (32, 33). Increasing SA concentrations induced lipotoxic damage in hepatocytes and skeletal muscle cells, and 250 μM SA induced endoplasmic reticulum stress in primary rat hepatocytes, leading to apoptosis (34, 35). In an animal model of insulin resistance, the ratio of palmitic acid to stearic acid was reduced from 3:1 in the normal group to 2:1 in mice fed a high-fat diet (36). In mouse primary pancreatic islet cells and cultured rat insulinoma INS-1E cells, SA inhibited insulin secretion by stimulating miR-34a-5p to suppress the expression of BCL2 and BCL-W, showing stronger lipotoxic effects than other fatty acids (37). Therefore, ratio of stearic acid contributes to cellular lipotoxicity as well, but additional in-depth studies on the molecular mechanisms associated with SA lipotoxicity are needed. The saturated fatty acid we used in the subsequent experiments was mainly PA because palmitic acid is widely used as a representative fatty acid for studying the relationship between SFAs and metabolic diseases and accounts for its highest proportion in dietary NEFAs in humans (38). In addition, the fatty acid concentrations used in our experiments were within the physiological concentration range (16, 39).

Many studies have reported the ameliorative effects of unsaturated fatty acids on metabolic diseases. For example, a low dose of 30 μM DHA at physiological or nutritional levels ameliorated palmitate (500 μM)-induced insulin resistance and the expression of inflammatory genes (*TNFα* and *IL-6*) in C2C12 cells (22). Similarly, in our experiments, UFAs ameliorated the SFA-induced impairments of cell viability, apoptosis, and ER stress. According to the literatures, co-treatments of 20 μM EPA, DHA, or AA with the ER stress agonist tunicamycin (TM, 2μg/mL) in the rat pancreatic islet β cells line RINm5F, AA did not reverse the TM-induced high expression of the apoptotic protein cleaved-caspase 3 or the ER stress marker proteins CHOP and XBP-1s (23). In our data, AA significantly reduced the protein levels of cleaved-caspase 3 as well as CHOP and XBP-1s in PA challenged INS-1E cells, but had no effect on IRE-1 or the BCL2/BAX ratio. This may be related to the differences in the concentrations of AA, as well as the different cell lines used.

SFAs induce ER stress, mitochondrial dysfunction, and oxidative stress causing lipotoxic damage. When unfolded or misfolded proteins accumulate in the endoplasmic reticulum, endoplasmic reticulum stress triggers the activation of the unfolded protein response (UPR), and BIP is released to activate PERK, IRE1α, and ATF6, as well as their downstream signaling pathways. Therefore, increased BIP expression is also considered an important indicator of ER stress (40). In mammals, ER calcium depletion, which results from alterations in the lipid composition of cell membranes, can promote unfolded protein stress by interfering with calcium-dependent chaperone proteins and enzymes required for protein folding, thereby activating the UPR through conventional sensing mechanisms (41, 42). Studies have shown that saturated free fatty acids deplete ER calcium ions and induce lipotoxic ER stress in pancreatic β cell lines, rat primary islet cells, and human islets. JNK and CHOP in the IRE1 and PERK pathways are involved in the execution of subsequent β-cell apoptosis, and the activation of ATF6 may have an antiapoptotic effect (31). In the present study, we observed PA-mediated activation of the IRE1α-XBP1 pathway in INS-1E cells, and PA-induced apoptosis is a failure of antiapoptotic regulation downstream of this pathway, ultimately, the cells undergo apoptosis. However, when we treated INS-1E cells with different FAs, we found that the protein level of BIP did not differ among the treatment groups, but the expression level of *Bip* mRNA was significantly increased in the PA-treated group. On the one hand, it may be that the expression of the BIP gene increased and the protein level was high when INS-1E cells were treated with a high concentration of PA, but the BIP protein was degraded rapidly, suggesting that there was no difference in the protein level among the groups. Another explanation for the activation of the UPR by disturbances in the lipid composition of cells is that excess free saturated fatty acid PA is sensed by the ER stress receptors IRE1α and PERK, which activate the UPR. Romain et al. showed that mutations in the stress-sensitive structural domains of the IRE1α and PERK luminal proteins maintain responsiveness to saturated lipids and activate the UPR, suggesting that the lipid environment directly regulates endoplasmic reticulum luminal stress-induced IRE1α and PERK activity, which may be two parallel pathways that intertwine with endoplasmic reticulum luminal stress (43).

Similarly, OA significantly ameliorated PA-induced ER stress in INS-1E cells. In an experiment in which pancreatic exocrine gland vesicle cells were treated with different concentrations of fatty acids (SFAs, MUFAs, and PUFAs) for 24 h, PA significantly increased the levels of UPR (XBP1, CHOP, and BIP) and cytokine (TNFα and TGFβ) transcripts, as well as enhanced the time-dependent nuclear translocation of XBP1. PUFAs (DHA) cause a milder increase in endoplasmic reticulum stress markers, whereas MUFAs (OAs) attenuate the endoplasmic reticulum stress response (44). Although UFAs are not detrimental to cell viability and ameliorate ER stress, excess unsaturated fatty acids lead to increased lipid deposition in cultured hepatocytes, so balanced fatty acid intake is necessary to maintain lipid homeostasis (45).

In our study, a low dose fish oil diet failed to improve insulin resistance in high-fat diet-induced obese mice, but FO had a superior modulatory effect on blood lipids compared to OO diet. In agreement with our results, the replenishment of FO or OO (60 mL/kg) to an atherogenic HFD significantly reduced plasma cholesterol levels in LDL receptor KO mice. However, FO significantly reduced atherosclerotic lesion area and NAFLD scores associated with steatosis and inflammation, whereas OO did not (46). A meta-analysis of 48 randomized controlled trials (RCTs) suggested that N-3 PUFA supplementation significantly reduces TG and TC levels in patients with metabolic syndrome (MetS) and related cardiovascular disease (CVD) (47). Interestingly, in our high-dose FO and OO high-fat dietary intervention experiments, both OO, and FO ameliorated hyperglycemia and hyperinsulinemia, increased insulin sensitivity, and modulated dyslipidemia in obese mice. However, only OO inhibited high-fat-induced compensatory islet hyperplasia. In a study by Enrique et al, C57BL/6 mice were first fed a HFD (45% lard) to induce T2D, and subsequently fed one of three different HFDs (lard, extra virgin olive oil (EVOO), or phenolic-rich EVOO) for 24 weeks. In this study, EVOO completely replaced the fatty acid supply in lard, and the HFD-EVOO significantly improved blood glucose levels, hyperinsulinemia, impaired glucose tolerance, and enhanced insulin sensitivity in T2D mice. In addition, the EVOO-related diet reduced β cell apoptosis and increased β cell mass, normalizing pancreatic glucose metabolism and glucose-induced insulin secretion. No other effects were observed at higher levels of phenolic compounds (48). These findings suggested that the polyphenols in OO do not contribute as much as other substances in OO, such as oleic acid, to improve blood glucose and enhance insulin sensitivity. Our previous results showed that PA impaired cell viability and insulin secretion of INS-1E cells and rat islets, but OA robustly rescued cells from cell death. OA substantially alleviated either PA or chemical ER stressors-induced ER stress. *In vivo*, an HFD for 32 weeks obviously induced islets ER stress and insulin resistance in SD rats. Half replacement of HFD with OO has ameliorated this effect and lowered FBI levels (6). Alkhatib et al. showed that OO intake prevents inflammation and oxidative stress in pancreatic β cell and improves β cell function and insulin resistance (49). In a pilot study conducted in Spain, participants were randomly assigned to receive a Mediterranean diet (MD) supplemented with EVOO, an MD supplemented with nuts, or a control low-fat diet without intervention; after 4.1 (median) years of follow-up, the risk of T2D onset was 40% lower in the group receiving MD+EVOO than in the control diet, whereas there was no statistically significant difference in the group receiving MD+nuts (50). In our experiment, the low-dose intervention experiment (OO) only had an ameliorative effect on IPITT. However in the high-dose intervention experiment, OO had a modulating effect on FBG and FBI in obese mice and also modulated overall insulin sensitivity (IPGTT and IPITT). The above suggests that OO reduces the risk of T2D development under normal physiological conditions. Under pathological conditions, it has the effect of improving blood glucose and Insulin levels, but the effect of the intervention is highly related to its dose.

OA also acts on peripheral insulin target organs such as the liver, muscle, and adipose tissue. Our previous study found that OA substantially alleviated PA-induced cellular apoptosis, oxidative stress, ER stress, mitochondrial dysfunction, as well as inflammation in hepatocytes *in vitro*. *In vivo*, HFD with olive oil has ameliorated non-alcoholic steatohepatitis (NASH) injury as well (33). Furthermore, G-protein-coupled receptor 3 (GPR3), which is associated with energy expenditure and obesity, is an attractive target for the treatment of metabolic disorders such as obesity and diabetes. Recent studies have shown that OA activates Gs/cAMP/PKA signaling in brown adipose tissue via GPR3 to drive thermogenesis in response to cold stimulation (51). Komiya et al. showed that dietary OO intake improved running endurance by increasing muscle triacylglycerol (TAG) accumulation in mice (52). Furthermore, OA improves mitochondrial maximal respiration and increases type 1 fiber formation in myotubes differentiated from C2C12 myoblasts (53). Mice were fed a 10% OA diet for 4 weeks. Yusuke et al. found that dietary OA intake improved running endurance and altered the fiber type composition of muscles, the proportion of type 1, and 2X fibers increased in the soleus muscle and type 2X increased in the EDL muscle (54). Overall, the effect of OA interventions is a combination of improvements on multiple fronts. On the one hand, attenuating islet lipotoxicity and improving islet function. On the other hand, it improves insulin resistance and energy metabolism in peripheral tissues. Its ameliorative effect is related to its anti-inflammation, reducing oxidative stress, anti-apoptosis, and reducing ER stress.

In addition, in our experiments, we found that high doses of FO elevated the serum liver damage index AST (**Supplementary Figure 5**). Therefore, after comparing the ameliorative effects of high and low doses of the unsaturated fatty acids FO and OO, respectively, in obese mice, we comprehensively recommend the OO diet as a dietary guideline for improving impaired glucose tolerance and islets function in obese and pre-diabetes patients.

## 5 Conclusion

In conclusion, both MUFAs and PUFAs can effectively protect islet β cells from SFA-induced cellular lipotoxicity, and their alleviation of cell apoptosis, ER stress and inflammation are the key mechanisms involved in their protective effects. Especially, OA showed the best protection among them, particularly on ER stress. In HFD induced obese mice, we demonstrated that low dose (5% energy replacement) FO or OO diet had limited effects, but high dose (20% energy replacement) FO and OO diets showed excellent improvements on insulin resistance and glucose-lipid metabolism. Particularly, fish oil has more potential on alleviating hyperlipidemia, but might have negative effect on liver function; while olive oil showed superior advantages on protecting islets function and insulin sensitivity. In summary, in comparison with PUFAs, MUFAs might have greater potential in nutritional intervention for pre-diabetic and diabetic individuals.

## Data availability statement

The raw data supporting the conclusions of this article will be made available by the authors, without undue reservation.

## Ethics statement

The animal study was approved by the experimental procedures were approved by the Institutional Animal Care and Use Committee (IACUC) of Sichuan University (Approval number: 20220119002). The study was conducted in accordance with the local legislation and institutional requirements.

## Author contributions

WL: Conceptualization, Data curation, Formal analysis, Investigation, Visualization, Writing – original draft, Methodology, Software, Validation. MZ: Formal analysis, Data curation, Writing – original draft. JL: Formal analysis, Data curation, Writing – original draft. SS: Formal analysis, Data curation, Writing – original draft. XZ: Formal analysis, Data curation, Writing – original draft. FF: Formal analysis, Data curation, Writing – original draft. YL: Project administration, Supervision, Resources, Writing – original draft. ZR: Project administration, Resources, Supervision, Writing – review & editing, Methodology. YC: Funding acquisition, Project administration, Resources, Supervision, Writing – review & editing, Methodology, Software.
