# Supplementary material for: Comparison of the effects of monounsaturated fatty acids and polyunsaturated fatty acids on the lipotoxicity of islets
Source: Front Endocrinol (Lausanne). 2024 Mar 4;15:1368853. doi: 10.3389/fendo.2024.1368853 (PMC10945794; doi:10.3389/fendo.2024.1368853)
Supplement: Supplementary file 1 [file DataSheet_1.pdf]

Figure S1

Low-dose unsaturated fatty acid diet intervention experiment

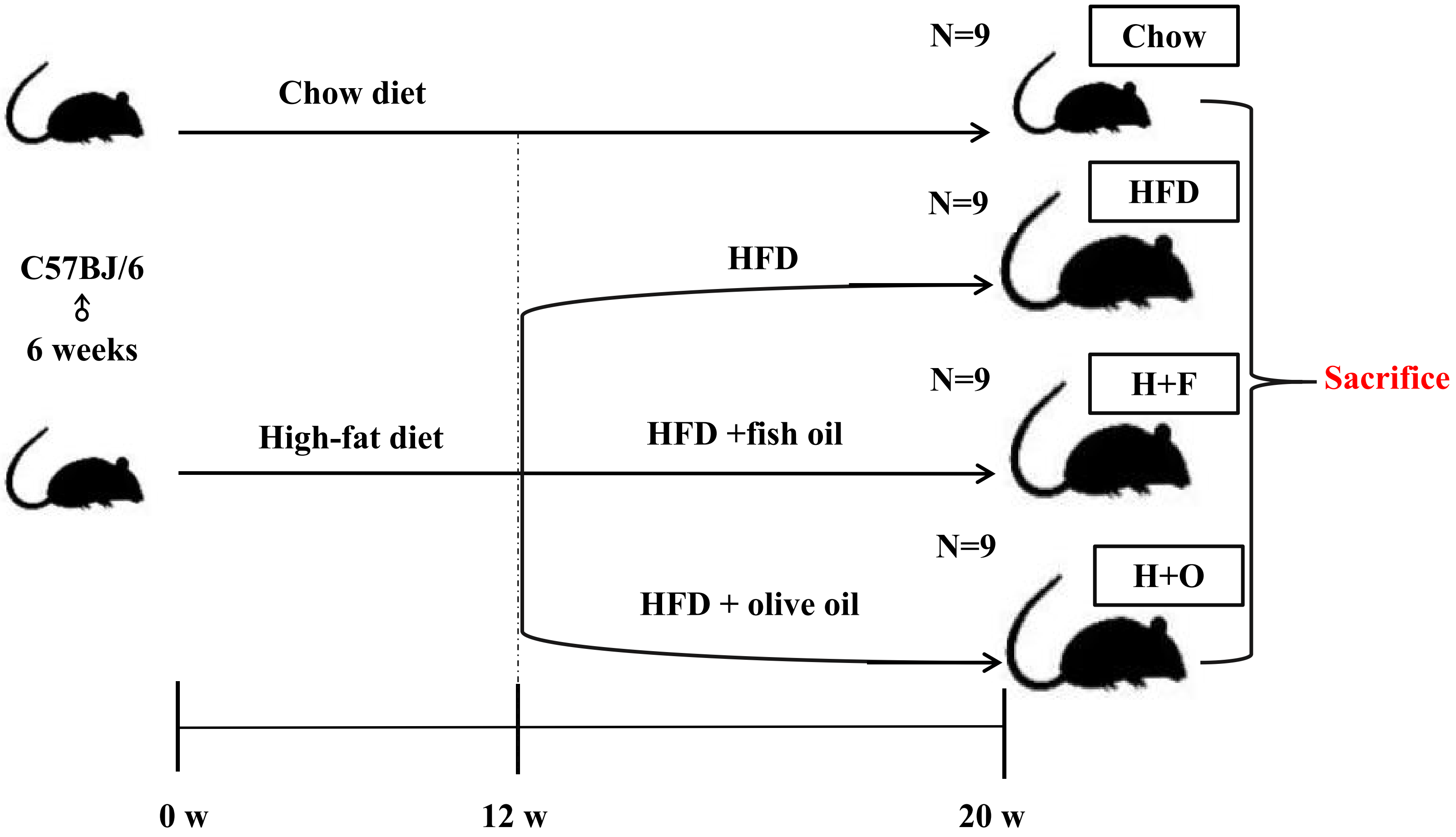

Figure S2

High-dose unsaturated fatty acid diet intervention experiment

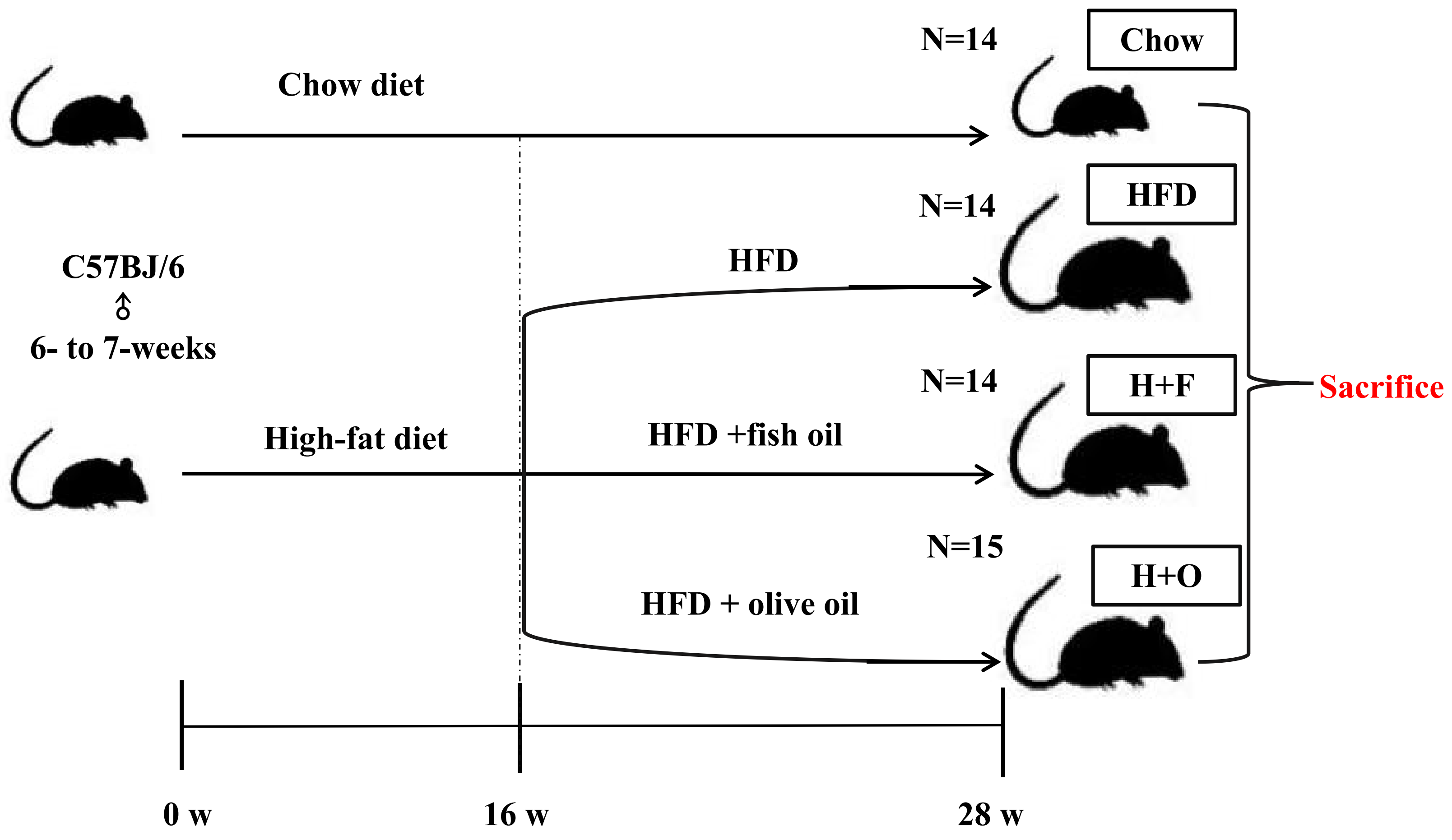

Figure S3

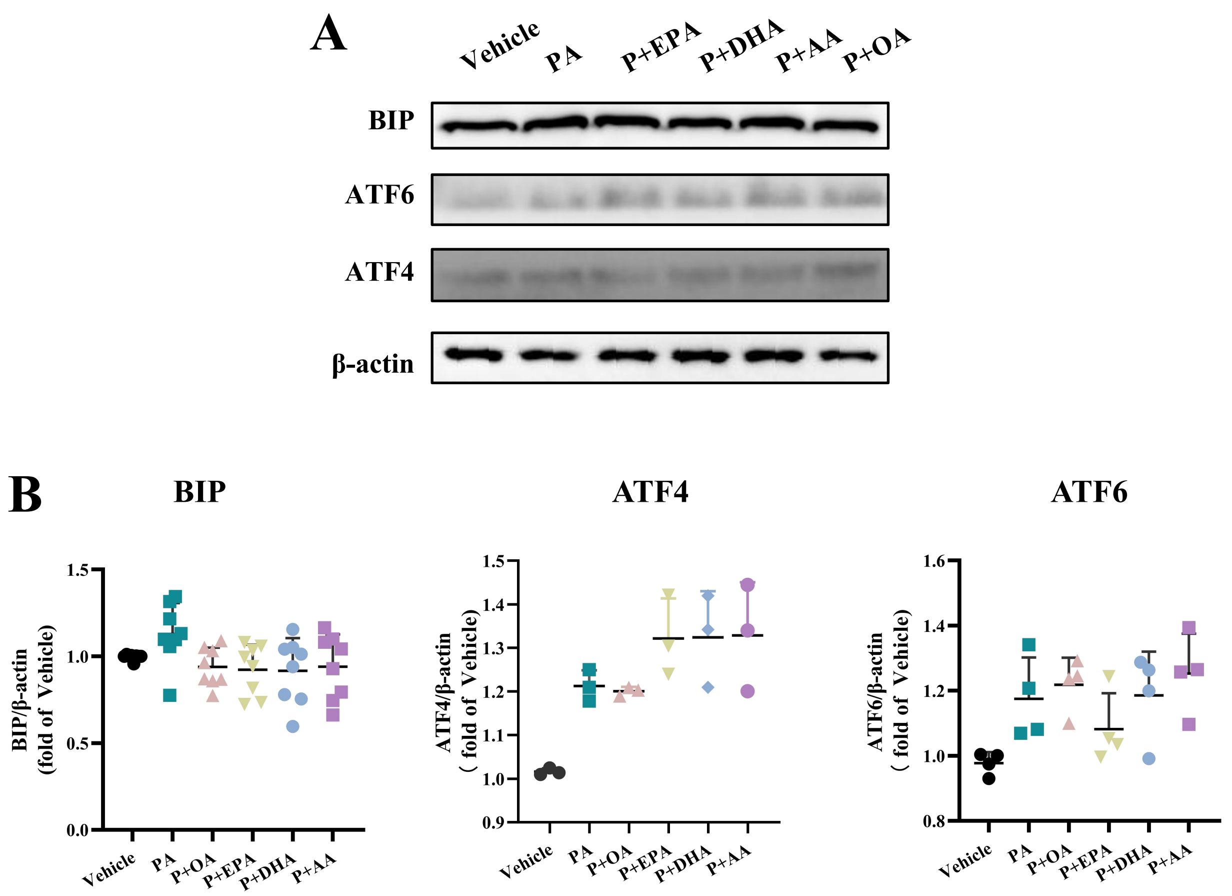

Figure S3. MUFAs/PUFAs ameliorate PA-induced ER stress and the expressions of inflammatory genes.
(A) The expression of ER stress markers (BIP, ATF4, and ATF6) were examined using immunoblots.
(B) Statistical analysis of protein expression levels in Figure A. Data are expressed as mean ± SD, (n≥3).

Figure S4

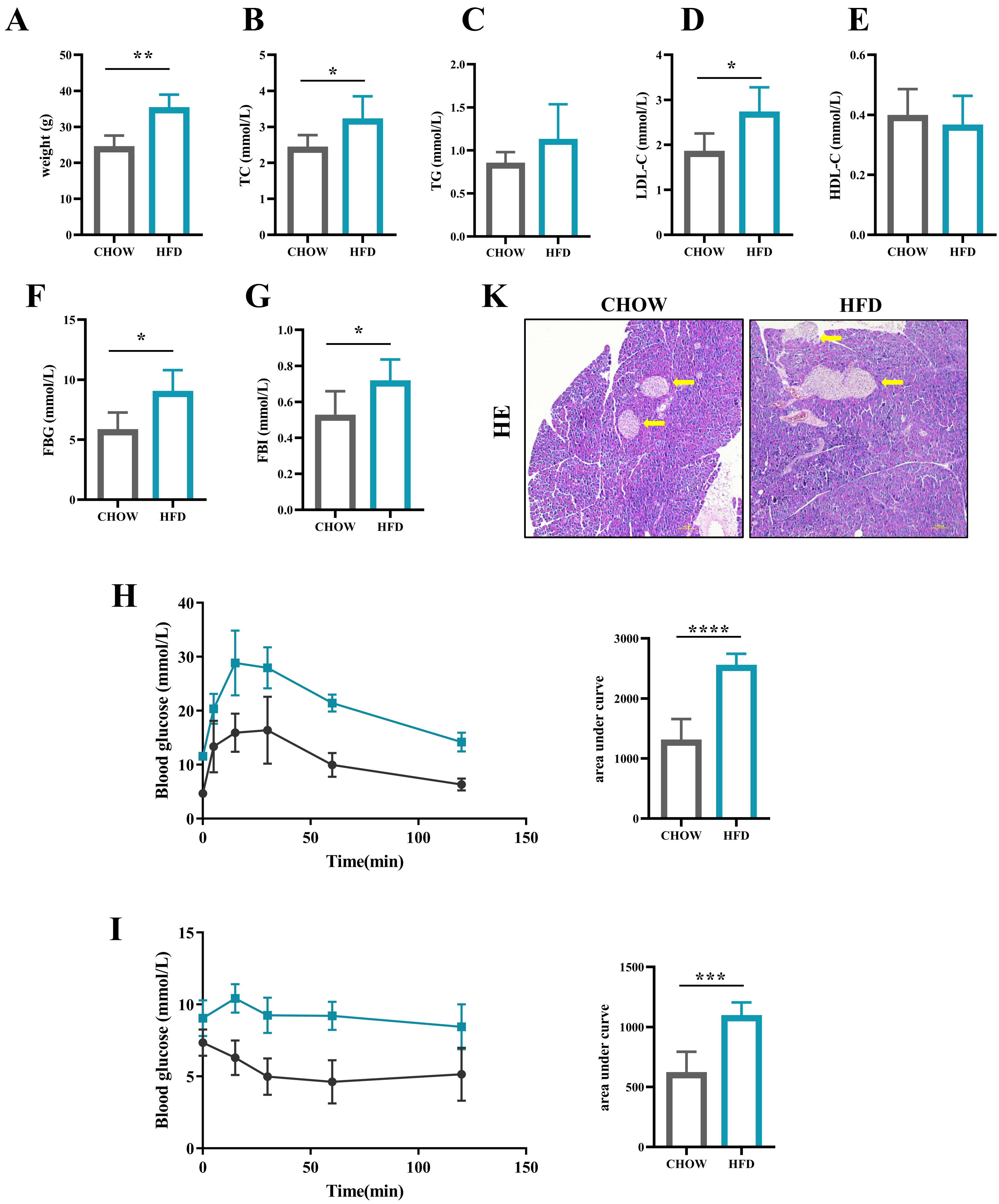

Figure S4. HFD feeding for 12 w induces obesity and insulin resistance in mice.
(A) Weight of mice. (B) TC. (C) TG. (D) LDL-C. (E) HDL-C. (F) FBG. (G) FBI. (H) IPGTT. (I)IPITT. (K) HE staining of pancreatic tissue (100X) (The yellow arrows show islets). $^{*}P$<0.05, $^{**}P$<0.01, $^{***}P$<0.001, $^{****}P$<0.0001. Data are expressed as the mean ± SD, (n≥5).

Figure S5

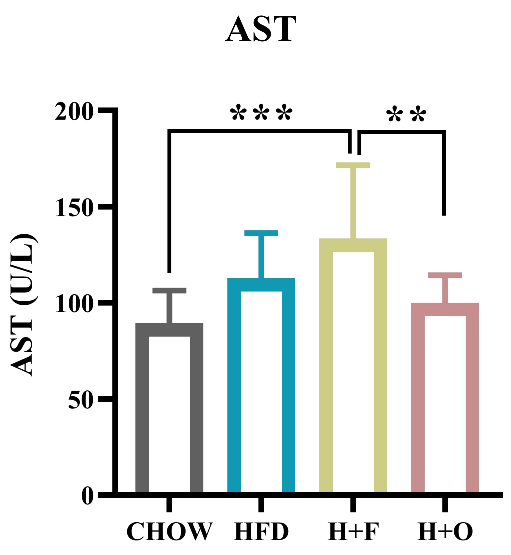

Figure S5. High-dose fish oil diets significantly increased AST levels in obese mice. (A) AST. **P<0.01, ***P<0.001. Data are expressed as the mean ± SD, (n≥5).

The details of the antibodies

Antibody	Manufacturer	Catalog:	Host species	Dilutions	Mol.Wt.:
Anti-Cleaved caspase-3 antibody	CST	9664S	rabiit	1:500	17
Anti-BAX antibody	CST	2772S	rabiit	1:500	20
anti-BCL-2 antibody	Abcam	ab196495	rabiit	1:500	26
anti-IRE1α antibody	CST	3294S	rabiit	1:400	130
anti-CHOP antibody	Affinity bioscience	DF6025	rabiit	1:500	19
Anti-actin antibody	ABclonal	AC026	rabiit	1:50000	42
anti-XBP-1 antibody	Wanlei	WL00708	rabiit	1:500	29/40
Anti-BIP antibody	Absin	abs130538	rabiit	1:500	75
Anti-peif2α antibody	CST	3398S	rabiit	1:500	38
anti-ATF4 antibody	Huaan	ET1612-37	rabiit	1:1000	55
anti-ATF6 antibody	Affinity bioscience	DF6009	rabiit	1:500	75
Anti-Insulin antibody	Huaan	Em80714	mouse	1:500	
anti-glucagon antibody	Absin	abS124780	rabiit	1:200	
Donkey Anti-Rabbit IgG H&L (DyLight® 550)	Abcam	ab96892	Donkey	1:200	
Donkey Anti-Mouse IgG H&L (Alexa Fluor® 488)	Abcam	ab150105	Donkey	1:200	
HRP labelled goat anti-rabbit IgG	Wanlei	WLA023	goat	1:4000	

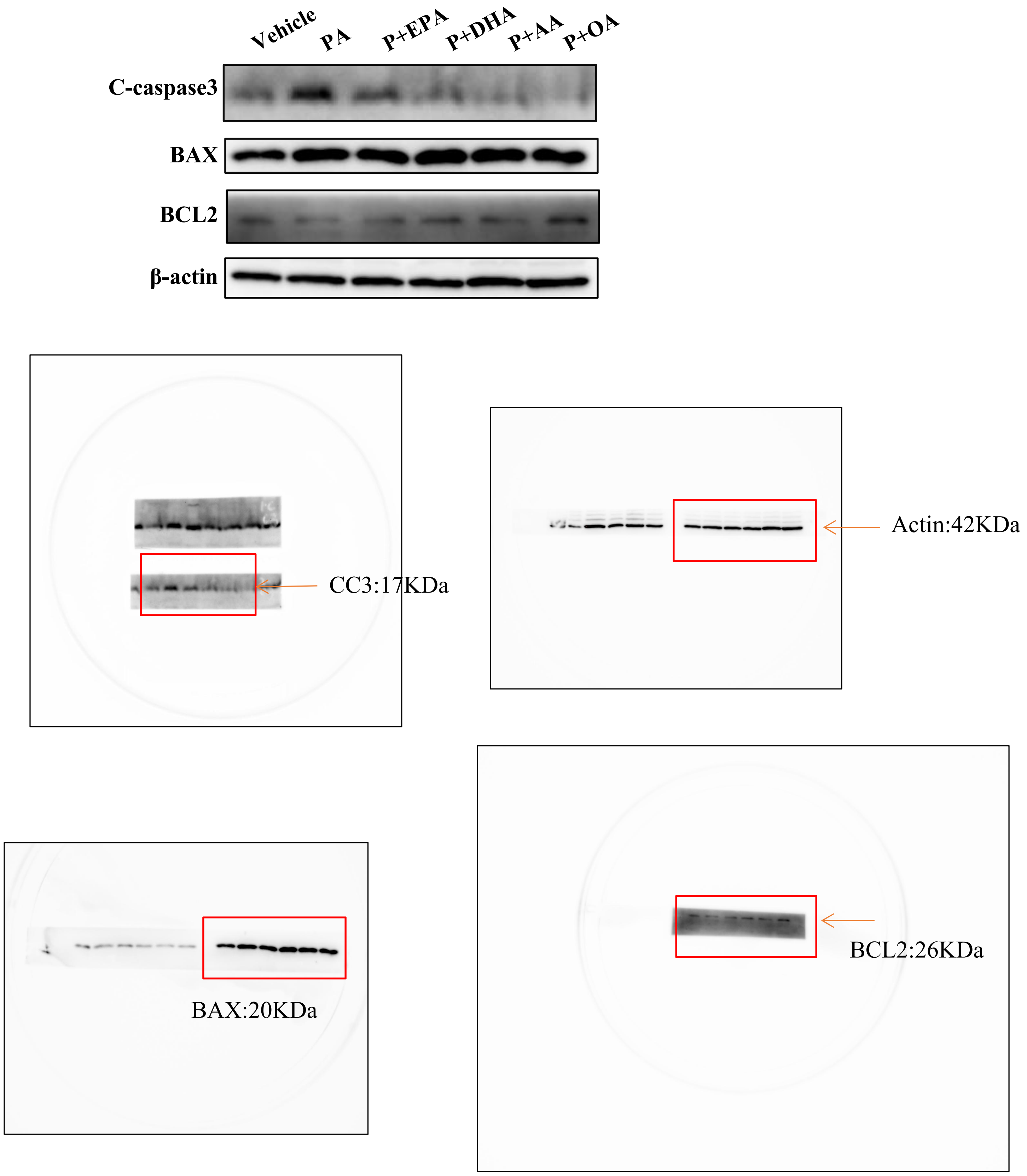

Vehicle
PA
P+EPA
P+DHA
P+AA
P+OA
C-caspase3
BAX
BCL2
β-actin
CC3:17KDa
Actin:42KDa
BAX:20KDa
BCL2:26KDa

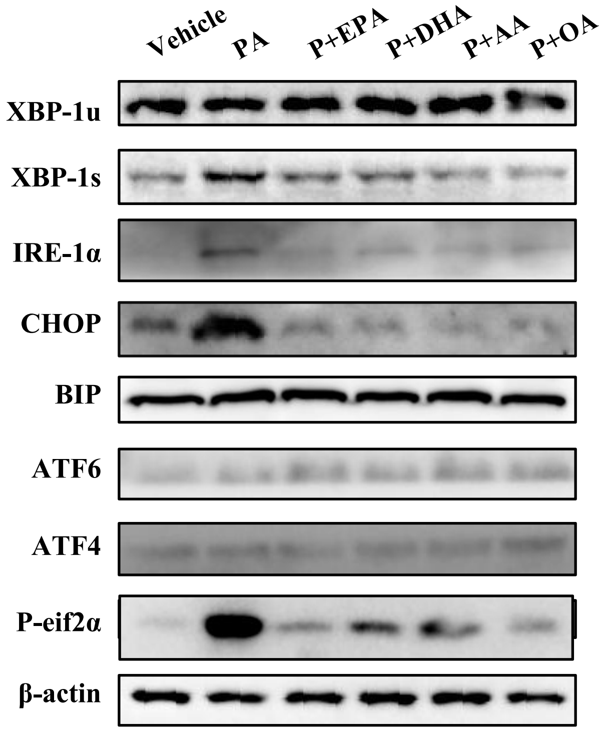
Vehicle
PA
P+EPA
P+DHA
P+AA
P+OA
XBP-1u
XBP-1s
IRE-1α
CHOP
BIP
ATF6
ATF4
P-eif2α
β-actin

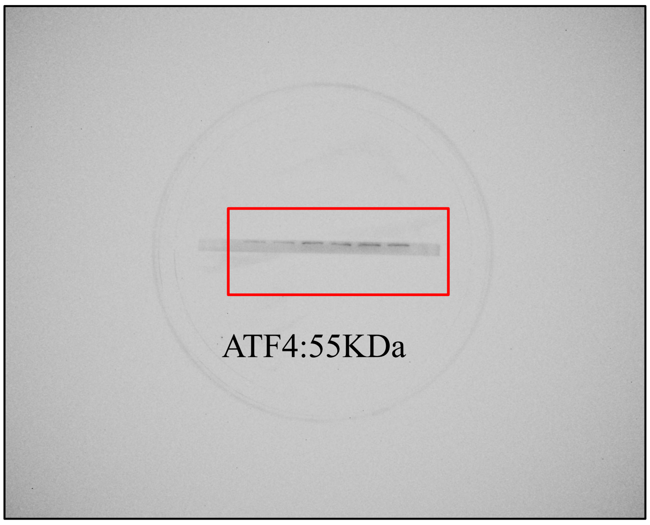
ATF4:55KDa

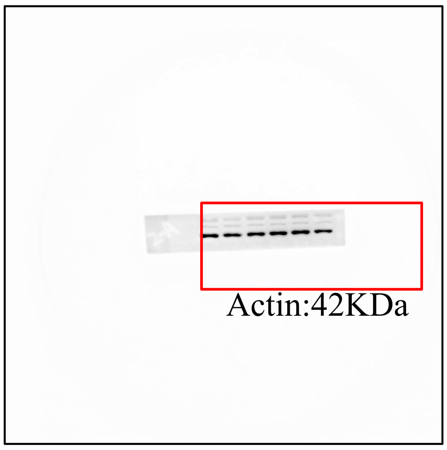
Actin:42KDa

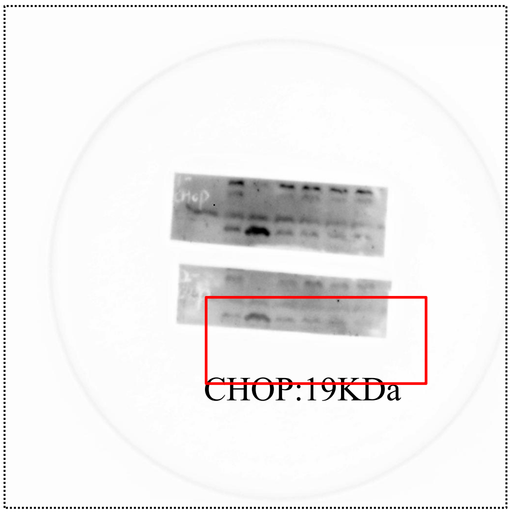
CHOP:19KDa

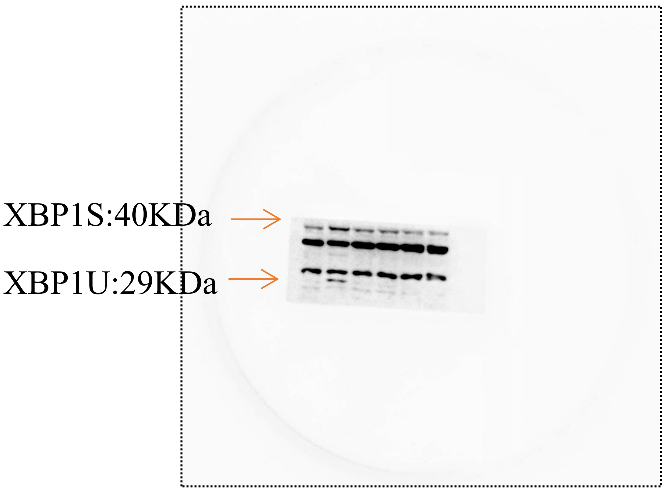
XBP1S:40KDa
XBP1U:29KDa

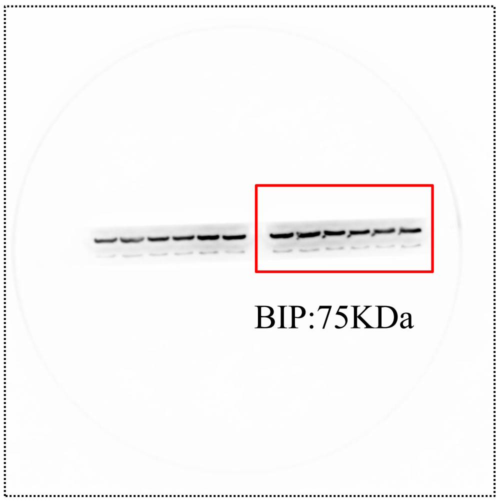
BIP:75KDa

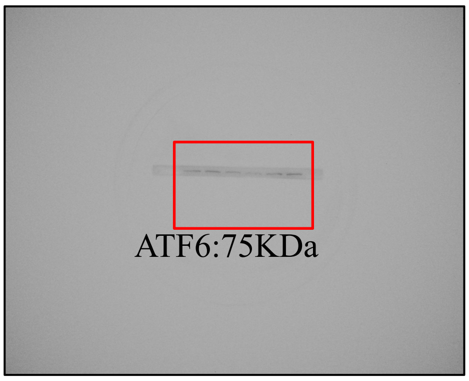
ATF6:75KDa

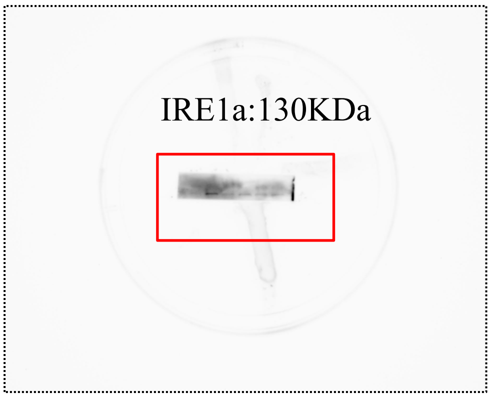
IRE1a:130KDa

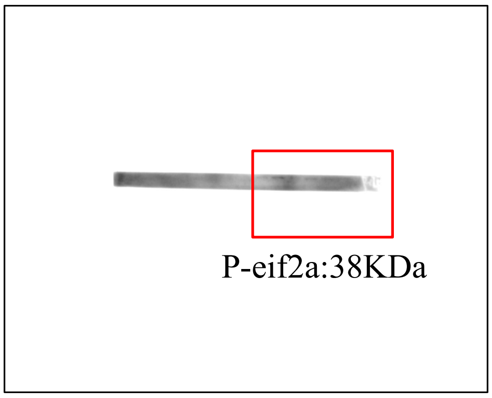
P-eif2a:38KDa

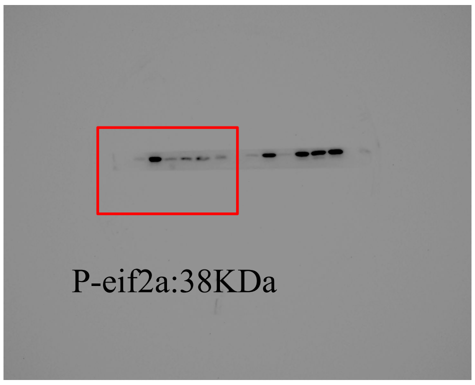
P-eif2a:38KDa
